# PAIN AND DEPRESSION AFFECT SELF-REPORTED STRESS RATINGS IN ADULTS WITH AND WITHOUT FIBROMYALGIA

**DOI:** 10.1093/geroni/igz038.2626

**Published:** 2019-11-08

**Authors:** Ha M Nguyen, Kristen Phillips, Barbara J Cherry, Laura Zettel-Watson

**Affiliations:** California State University, Fullerton, Fullerton, California, United States

## Abstract

Fibromyalgia (FM) is a chronic pain condition characterized by tenderness, fatigue, stiffness, joint pain, and physical and emotional distress. Depression is common, as well, and stress may be perceived as more severe. The current study examines perceived stress in adults ages 50 and older with and without FM. It was hypothesized that individuals with FM and/or depression would subjectively rate stressors as more severe compared to those without. Ninety-four participants (53% with FM, 78% female) aged 50 to 93 (M = 67.72, SD = 9.26) were administered an updated version of the Social Readjustment Rating Scale (SRRS) to assess amount of stress experienced in the past year. The difference between the SRRS pre-determined values and participants’ subjective ratings was calculated. Lower difference scores indicated that self-reported severity exceeded standardized values. Hierarchical linear regression analyses revealed that older adults and men were less likely to report exaggerated stressor severity (p < .05). Controlling for age and gender, individuals with FM were significantly more likely to report stressor severity far above standardized severity scores (p < .05). Results also revealed that both depression and chronic pain impact stress ratings, but impact is significantly greater for depression (p < .001). When controlling for depression, FM impact is no longer significant, suggesting that depression is a stronger predictor of subjective stress. The findings emphasize the importance of stress management and lifestyle changes to reduce the likelihood of depression and stress perception in individuals experiencing chronic pain.

